# Synergies and trade-offs in forest carbon pools: separating universal drivers from forest type-specific controls

**DOI:** 10.3389/fpls.2025.1728262

**Published:** 2026-01-06

**Authors:** You Zhou, Jiyun She, Guohong Xiang, Xiongmei Zhu

**Affiliations:** 1 College of Forestry, Central South University of Forestry and Technology, Changsha, China; 2 Hunan University of Humanities, Science and Technology, Loudi, China

**Keywords:** biodiversity, carbon allocation, natural forest, subtropical forest, synergy and trade-off

## Abstract

**Introduction:**

Understanding the synergies and trade-offs among tree, understory, and soil carbon pools is critical for optimizing forest carbon sinks. However, the mechanisms regulating these relationships, particularly how they differ between natural and planted forests, remain unclear. This study aims to deconstruct these complex interactions in subtropical forests to provide a scientific basis for enhancing ecosystem carbon storage.

**Methods:**

Based on data from 440 plots covering six major forest types in subtropical China, we employed linear mixed-effects models (LMMs) to quantify universal and context-dependent driver effects. We then used structural equation models (SEMs) to test and compare the mechanistic pathways of carbon allocation in natural versus planted forests.

**Results:**

Our LMMs revealed a universal trade-off, with tree layer carbon density (TCD) strongly suppressing understory carbon density (UCD) (β = -0.22, P < 0.001) while synergistically promoting soil organic carbon density (SOCD) (β = 0.36, P < 0.001). SEM analysis (natural forests: CFI = 0.986, RMSEA = 0.064; planted forests: CFI = 0.960, RMSEA = 0.076) revealed divergent regulatory mechanisms. In natural forests, tree diversity directly buffered the suppressive effect of TCD on UCD via a significant positive path (β = 0.22, P < 0.01). This buffering pathway was absent in planted forests, leading to an amplified TCD-UCD trade-off (β = -0.54, P < 0.001).

**Discussion:**

Our findings demonstrate that forest carbon allocation is governed by a vertical trade-off and an above-belowground synergy, with tree diversity acting as a key modulator. Compared with complex natural forests, the carbon allocation mechanism in planted forests is simplified with more acute trade-offs. We conclude that enhancing structural and species diversity in plantations is a critical pathway for synergistically optimizing the entire ecosystem’s carbon sink capacity.

## 1 Introduction

Forest ecosystems are central to the global biogeochemical cycle and regulate considerable carbon fluxes between the land and atmosphere (Bréchet et al., 2018). As the largest terrestrial carbon reservoir, forests account for more than half of the total carbon found in global vegetation and soils (Vos et al., 2020). Consequently, a persistent net change in their carbon sequestration capacity, even if small in annual terms, can accumulate over time and significantly influence the trajectory of future climate change (IPCC, 2021). A fundamental scientific challenge, therefore, is to accurately quantify forest carbon storage and understand the complex mechanisms, such as carbon allocation, inter- and intraspecific competition, and litter decomposition, that regulate this process. This understanding is essential for developing effective nature-based strategies to enhance carbon sinks in the context of ongoing climate change. Forest carbon storage comprises the following two primary pools: aboveground carbon (AGC) in biomass and belowground soil organic carbon (SOC). These pools are closely coupled through ecological processes such as litter input, root turnover, and microbial activity, resulting in the formation of a dynamic continuum (Schmidt et al., 2011).

Previous research has established a robust framework that identifies climate, topography, and stand attributes as the primary drivers of forest carbon storage (Reta et al., 2025; Wu et al., 2025). However, these studies have often relied on a reductionist approach and have focused on investigating the drivers of above- and belowground carbon pools independently. This perspective largely does not account for the intrinsic connections that define an ecosystem as an integrated system. Consequently, a critical question remains unanswered: do factors that benefit the aboveground component, such as the tree layer, also synergistically promote other parts of the system, such as the understory and soil (Gamfeldt et al., 2013)? In fact, trade-offs in resource allocation are common among ecosystem components (Ren et al., 2014). For instance, a widely accepted hypothesis states that a dense tree canopy suppresses understory growth through asymmetric competition (Heiland et al., 2023; Markgraf et al., 2023). This vertical trade-off may then cascade to affect the long-term accumulation of soil carbon (Vesterdal et al., 2013; Makoto et al., 2022).

Although this vertical trade-off is common, its regulatory mechanisms and potential mitigation pathways remain key knowledge gaps in ecology. One important but not fully validated hypothesis is that biodiversity serves as a buffer. Theoretically, high species diversity can facilitate resource use optimization and an improvement in the understory microenvironment through niche complementarity and facilitation. This, in turn, could alleviate the suppression of the understory while simultaneously enhancing tree layer productivity (McNicol et al., 2017; Fan et al., 2020; Yan et al., 2024). However, whether this diversity-mediated buffering effect occurs and whether its magnitude depends on the broader ecosystem context require testing with large-sample, real-world data.

Forest origin and management (natural vs. planted forests) are key factors that define this context. Natural and planted forests are distinct ecosystems in terms of their structure, function, and successional stage, providing an excellent platform for testing these hypotheses (Gdula et al., 2021). Compared with planted forests, natural forests typically exhibit more complex vertical structures, greater diversity, and more stable internal regulatory mechanisms, which may lead to weak trade-offs (Cheng et al., 2021, Cheng et al., 2024; Lv et al., 2025). In contrast, the simplified structure and low species richness of planted forests may intensify competition and result in greater trade-offs (Carnus et al., 2006; Peris-Llopis et al., 2024).

Therefore, using multilevel carbon pool data from diverse natural and planted forests in subtropical China, this study aimed to deconstruct the pathways governing carbon allocation and test the context-dependency of these mechanisms. Specifically, we sought to: 1) identify the direct and indirect pathways through which drivers of tree layer carbon (TCD) affect understory (UCD) and soil (SOCD) carbon pools, and determine whether these interactions result in synergies or trade-offs; and 2) evaluate whether these cross-level relationships differ between natural and planted forests. We hypothesized that stand structure, particularly tree diversity, is the primary mechanism regulating these relationships, and that its buffering effect on the tree-understory trade-off is substantially stronger in natural forests than in planted forests.

## 2 Materials and methods

### 2.1 Study area

Our study was conducted in the southern–central region of Hunan Province, China (24°39′–28°14′ N, 110°27′–114°14′ E), which is located in the mid-subtropical evergreen broad-leaved forest biome. The region has a typical humid subtropical monsoon climate with favorable hydrothermal conditions and four distinct seasons. On the basis of meteorological data from the past 30 years, the mean annual temperature (MAT) is 16.5 °C, and the mean annual precipitation (MAP) reaches 1500 mm. The geomorphology is spatially heterogeneous, consisting of northeast–southwest trending mountain ranges (e.g., the Xuefeng and Luoxiao Mountains) interspersed with hills and basins. This topography creates a broad elevational gradient from 50 to 1547 m.

The combination of diverse hydrothermal conditions and a complex topographic gradient supports rich forest ecosystems. The primary vegetation types include natural evergreen broad-leaved secondary forests, dominated by species from the Lauraceae and Fagaceae families. The region also encompasses extensive planted forests established for timber production. These plantations are primarily monocultures of Chinese fir (*Cunninghamia lanceolata*) and Masson pine (*Pinus massoniana*) but also include some planted broad-leaved and mixed broadleaf-conifer stands. As a crucial ecological barrier and a major timber production base in southern China, the area is subject to complex interactions between anthropogenic activities and conservation efforts. The area’s diverse forest management models and wide environmental gradients provide an ideal natural laboratory for testing the context-dependency of carbon allocation mechanisms.

### 2.2 Plot design and field sampling

Study plots were established in 2021 via a stratified random sampling strategy (Cochran, 1977) that integrated geographic information system (GIS) data and national forest inventory data. First, we divided the study area into multiple strata on the basis of vegetation type, altitude (from a DEM), and stand age. Sample points were then randomly generated within each stratum in proportion to its area. From these points, we established 440 permanent 25.82 m × 25.82 m square plots (approx. 667 m², which is equivalent to one mu in the Chinese national forest inventory system). This design ensured comprehensive and unbiased coverage of the six major forest types (natural broad-leaved, natural coniferous, natural broadleaf-conifer mixed, planted broad-leaved, planted coniferous, and planted broadleaf-conifer mixed) across their full range of stand ages and topographic gradients. The spatial distribution of all 440 plots across the study area is provided in the **Supplementary Material (Figure A1)**.

**Figure 1:**
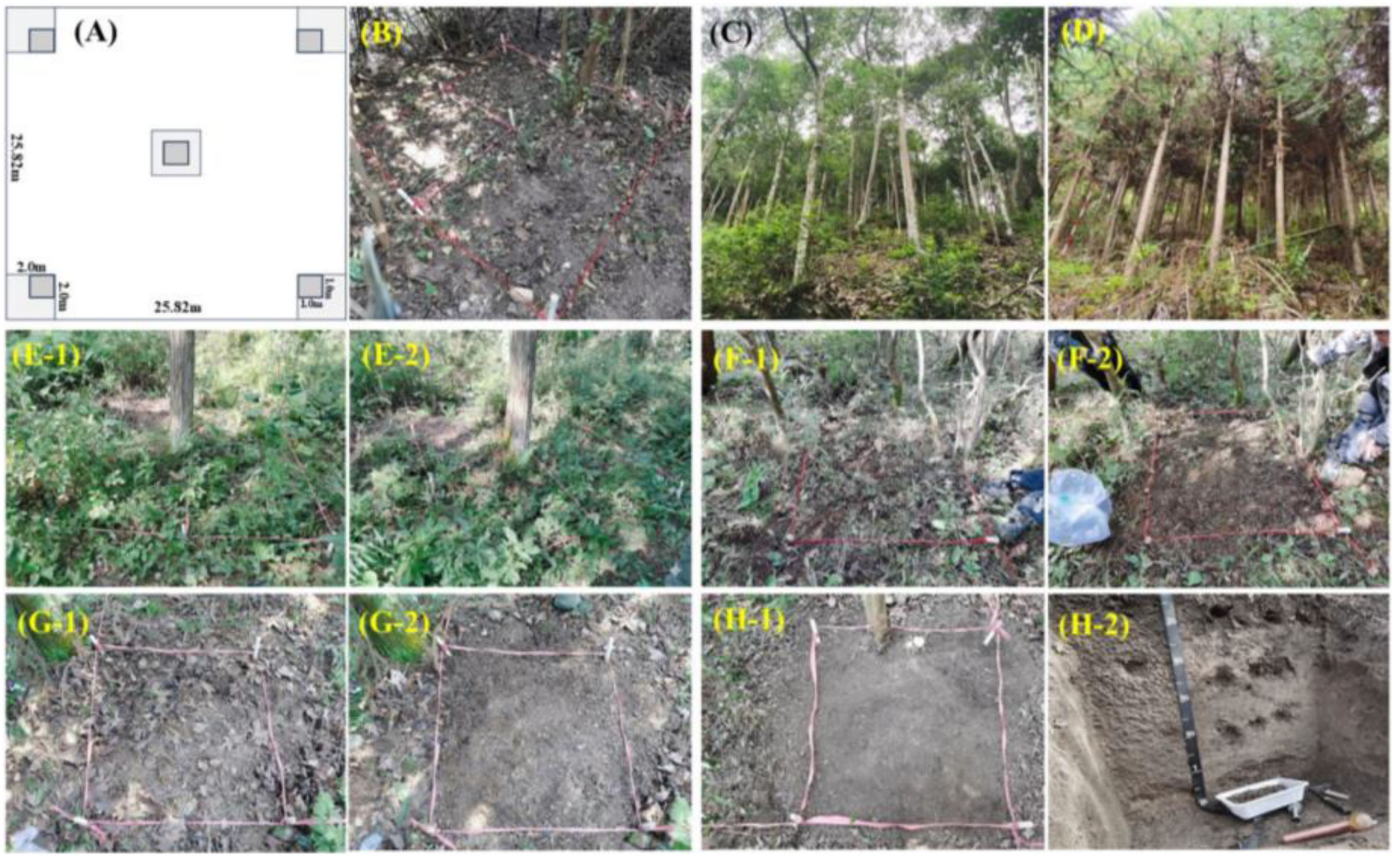
Multilevel nested sampling design used in this study. **(A)** Schematic of a 25.82 m × 25.82 m plot, showing the five 2 m × 2 m shrub quadrants with their nested 1 m × 1 m subplots. **(B)** Field view of a shrub quadrat. **(C, D)** Representative canopies of a natural broad-leaved forest and a planted coniferous forest. **(E-1, E-2)** Harvesting the shrub biomass. **(F-1, F-2)** Delineating an herb–litter subplot. **(G-1)** Litter layer before removal. **(G-2)** Sampling the herbaceous layer after litter removal. **(H-1)** A soil sampling point with a soil auger. **(H-2)** A soil profile and collection of an undisturbed soil core.

Field sampling followed a standardized, multilevel nested protocol to ensure data comparability and accuracy (**Figure 1A**), as shown by photographs of typical forest conditions and field procedures (**Figure 1**).

Tree layer: In each plot, all trees with a diameter at breast height (DBH, 1.3 m) ≥ 5 cm were tagged and inventoried. We recorded the species, DBH, total height, crown width, height to live crown base, and health status of each tree.

**Figure 2:**
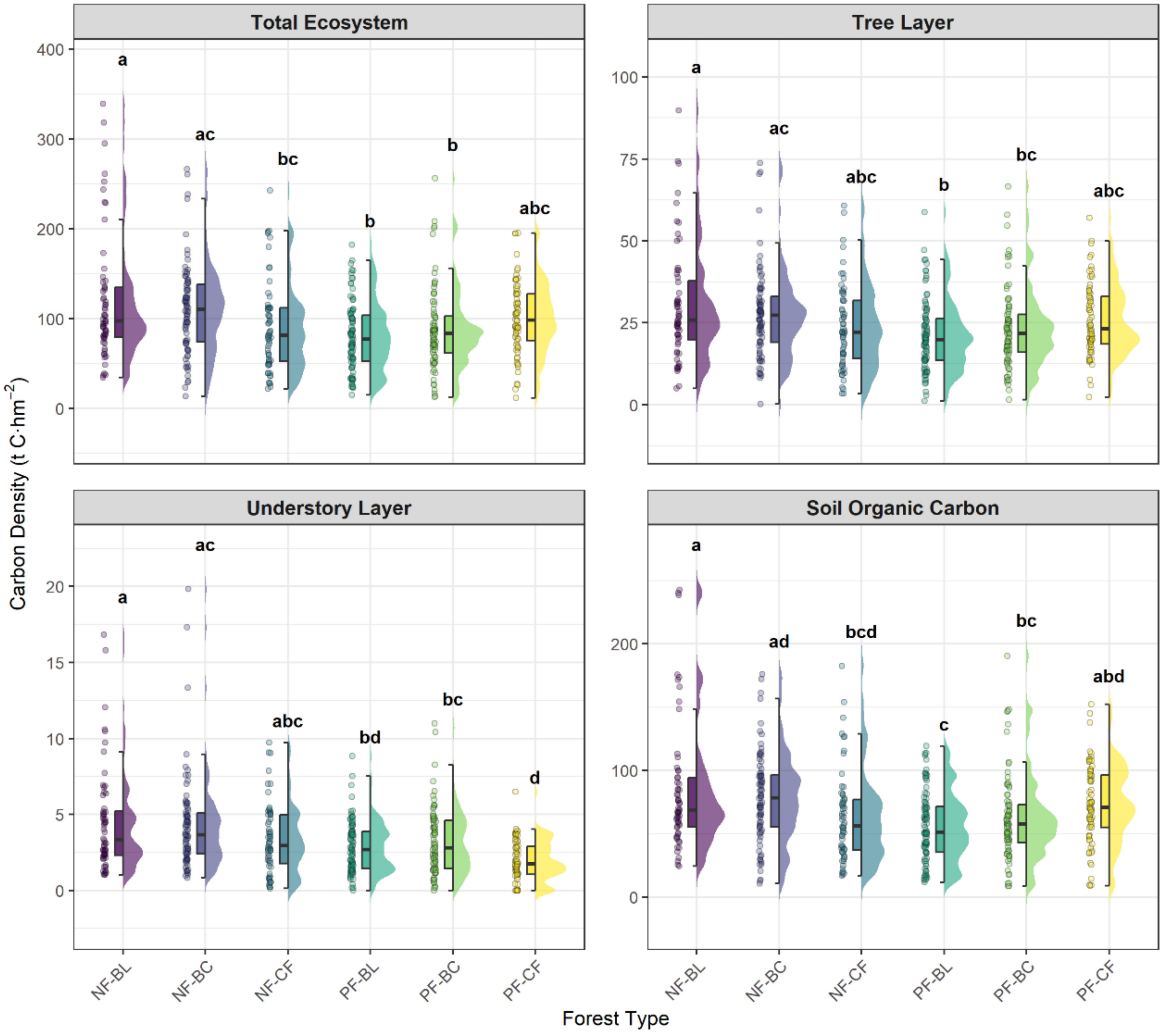
Carbon densities of the total ecosystem (TECD), tree layer (TCD), understory layer (UCD), and soil organic carbon (SOCD) across six subtropical forest types. The plots show the data distribution (half-violin), summary statistics (boxplots), and raw data points (jittered points). Different letters above the plots indicate significant differences among forest types according to Tukey’s honestly significant difference (HSD) test (*P* < 0.05).

Understory vegetation and litter: In each plot, we established five 2 m × 2 m shrub quadrants at the center and four corners (**Figure 1B**). In each quadrat, all the shrubs (woody-stemmed plants < 5 m tall) were identified to the species level, and their abundance, basal diameter, and mean height were recorded. Representative individuals were harvested to estimate aboveground biomass (**Figures 1E-1, 1E-2**). A 1 m × 1 m subplot for herbs and litter was nested within each shrub quadrat (**Figures 1F-1, 1F-2**). We first collected the entire litter layer, including undecomposed (L) and semidecomposed (F) material, and recorded its thickness (**Figure 1G-1**). We then identified all the herbaceous plants; recorded their species, cover, and abundance; and clipped all the aboveground parts (**Figure 1G-2**). All the harvested samples were sorted by type and bagged.

Soil layer: After the surface litter was removed, we collected soil samples from the center of each of the five subplots. A 5 cm diameter soil auger was used to sample four successive depths: 0–20 cm, 20–40 cm, 40–60 cm, and 60–100 cm (**Figure 1H-1**). For each depth, the five cores were thoroughly mixed to form one composite sample for chemical analysis. Concurrently, at each sampling point and for each layer, we also collected an undisturbed soil core using a 100 cm³ cutting ring to determine the bulk density and water content (**Figure 1H-2**).

### 2.3 Laboratory analysis and carbon stock calculations

All the collected samples were transported to the laboratory for processing. To determine their biomass, the plant samples (shrubs, herbs, and litter) were oven-dried at 65 °C to a constant weight. Soil samples were air-dried, and visible roots and gravel were carefully removed. A subsample of the soil was then ground to pass through a 0.25 mm sieve for chemical analysis. The soil organic carbon (SOC) content was determined using the potassium dichromate (K_2_Cr_2_O_7_) external heating–oil bath titration method (Matazu et al., 2024). Undisturbed soil cores were oven-dried at 105 °C to a constant weight to calculate the bulk density (BD) and gravel content. The carbon density in each pool was calculated as the carbon stock per unit area (t C·hm^-^²). The descriptive statistics for all the carbon pools are summarized in **Table 1**.

**Table 1:** Descriptive statistics of carbon densities for different ecosystem pools used in the analyses (N = 440).

Ecosystem pool	Abbreviation	Unit	Minimum	Maximum	Mean	STD
Measured Pools						
Tree Layer Carbon Density	TCD	t C·hm^-^²	0.26	89.76	25.05	13.32
Shrub Layer Carbon Density	SCD	t C·hm^-^²	0.14	12.68	2.36	1.92
Herbaceous Layer Carbon Density	HCD	t C·hm^-^²	0.02	7.15	1.27	1.01
Litter Layer Carbon Density	LCD	t C·hm^-^²	0.10	15.50	2.15	1.85
Soil Organic Carbon Density (0–100 cm)	SOCD	t C·hm^-^²	8.53	242.66	68.74	37.05
Calculated Pools						
Understory Layer Carbon Density	UCD	t C·hm^-^²	0.14	19.83	3.32	2.56
Total Ecosystem Carbon Density	TECD	t C·hm^-^²	11.9	339.07	97.12	50.64

STD denotes the standard deviation, and UCD is the sum of SCD, HCD and LCD. TECD denotes the sum of TCD, UCD, and SOCD.

Vegetation carbon was determined by converting biomass estimates to carbon stocks. The aboveground biomass (AGB) of individual trees was estimated using locally validated allometric equations, with diameter at breast height (DBH) and height (H) as the primary predictors (Standardization Administration of China, 2024). Shrub and herb biomass densities were extrapolated from the dry mass of the harvested samples. All biomass values were then converted to carbon stock using a conversion factor of 0.5 (Manning and Solomon, 2007).

The soil organic carbon (SOC) density for each soil layer (SOCD_i_) was calculated on the basis of the SOC content (SOC_i_, g·kg^-^¹), bulk density (BD_i_, g·cm^-^³), layer thickness (D_i_, cm), and volumetric gravel content > 2 mm (G_i_, %) as follows:

SOCD_i_ = SOC_i_ × BD_i_ × D_i_ × (1 - G_i_/100) × 0.1.

### 2.4 Explanatory variables

We selected 11 explanatory variables across three categories, i.e., topography, climate, and stand attributes, to investigate the drivers of carbon pool variation (**Table 2**). All the geospatial variables were extracted using the GPS coordinates of the sample plots in ArcGIS 10.8.

**Table 2:** Descriptive statistics of the environmental and stand-level explanatory variables used in the analyses (N = 440).

Category	Variable	Abbreviation	Unit	Minimum	Maximum	Mean	STD
Topographic	Altitude	Alt	m	219	1868	1054	357
	Slope	–	°	6	45	25	12
Climatic	Mean Annual Temperature	MAT	°C	12.0	26.0	19.0	4.2
	Mean Annual Precipitation	MAP	mm	1148	2250	1561	155
Stand Structure	Stand Density	SD	stems·hm^-^²	750	3750	1921	672
	Mean DBH	DBH	cm	5.6	32.0	17.6	7.0
	Mean Height	H	m	6.2	21.6	10.8	2.9
	Canopy Density	Canopy	-	0.50	0.90	0.70	0.11
Diversity	Tree Diversity (Shannon Index)	Tree_S	-	3.5	37.8	16.7	6.7
	Understory Diversity	Understory_S	-	5.3	30.1	16.9	5.1

STD stands for standard deviation. All diversity metrics were calculated using the Shannon–Wiener index.

Topographic factors were derived from the ASTER Global Digital Elevation Model Version 2 (ASTER GDEM V2) at a 30-m spatial resolution (NASA/METI/AIST/Japan Spacesystems, and U.S./Japan ASTER Science Team, 2011). From this DEM, we extracted the following two variables for each plot: altitude and slope. Climatic factors were sourced from the WorldClim 2 database, a high-resolution (~1 km²) gridded dataset based on interpolated weather station data from 1970–2000 (Fick and Hijmans, 2017). We extracted the MAT and MAP for each plot. Stand factors were derived from our field survey data. These included the categorical variable forest type (six levels; see Section 2.2) and several continuous variables. Stand structure was described by stand density (stems with DBH ≥ 5 cm per hectare), mean DBH, mean height, and canopy density. Community diversity was quantified using the Shannon–Wiener index (H’) (Shannon, 1948). This index was calculated separately for each plot. Tree diversity (Tree_S) was computed using the species identity and abundance (number of stems) of all the trees within the 25.82 m × 25.82 m plot. Understory diversity (Understory_S) was computed using the combined species and abundance data from all the shrub and herbaceous plants recorded across the five 1 m × 1 m subplots within each plot.

### 2.5 Statistical analysis

All the statistical analyses were performed in R (version 4.3.2; R Core Team, 2023) Our analysis followed a progressive workflow from pattern detection to mechanistic deconstruction.

First, we used one-way analysis of variance (ANOVA) with Tukey’s honestly significant difference (HSD) *post hoc* tests to compare the mean carbon densities (TCD, UCD, and SOCD) among the six forest types. Second, we calculated a Pearson correlation matrix for all the continuous variables to explore the relationships among them and to determine potential multicollinearity (Zuur et al., 2010).

The core of our analysis involved linear mixed-effects models (LMMs), which we used to quantify the independent effects of each driver while controlling for differences among forest types. We developed separate models for the three response variables, i.e., TCD, UCD, and SOCD, via the lme4 package (Bates et al., 2015). Before modeling, all the response variables were log-transformed (log(x+1)) to meet the assumption of normality, and all the continuous explanatory variables were standardized (z score transformation). Forest type was included as a random effect to account for data nonindependence and to distinguish between universal drivers (fixed effects) and context-dependent relationships (random effects). We selected the best-fit model using the Akaike information criterion corrected for small samples (AICc) (Burnham and Anderson, 2002), choosing the most parsimonious model when ΔAICc < 2.

Finally, to investigate causal mechanisms, we constructed structural equation models (SEMs) for natural and planted forests separately using the lavaan package (Rosseel, 2012). Model fit was evaluated using the chi-square test (χ² test, *P* > 0.05), the comparative fit index (CFI > 0.95), and the root mean square error of approximation (RMSEA < 0.08) (Kline, 2015). The significance level for all final models was set at *P* < 0.05.

## 3 Results and analysis

### 3.1 Carbon pool allocation and differences among forest types

Our study included 440 plots, comprising 200 natural forest (NF, 45.5%) and 240 planted forest (PF, 54.5%) sites. The mean total ecosystem carbon density (TECD) was 96.59 ± 50.98 t C·hm^-^² (**Table 3**). Carbon was unevenly allocated among the vertical strata: soil was the largest carbon reservoir, with a mean soil organic carbon density (SOCD) of 69.16 ± 40.33 t C·hm^-^² in the 0–100 cm layer, accounting for 71.6% of the total ecosystem carbon stock. In contrast, the mean aboveground carbon (AGC) density reached 27.43 t C·hm^-^². Within the AGC, the tree layer carbon density (TCD) was the dominant component (91.3%), with a carbon density of 25.05 ± 13.32 t C·hm^-^², whereas the understory layer carbon density (UCD) contributed only a minor amount at 2.37 ± 1.54 t C·hm^-^² (**Table 3**).

**Table 3:** Descriptive statistics of carbon pools (mean ± standard deviation, t C·hm^-^²) across six subtropical forest types (N = 440).

Forest type	N	Tree layer C density	Understory C density	Soil organic C density	Total ecosystem C density
NF-BC	80	27.69 ± 13.63	2.55 ± 1.16	78.89 ± 40.21	109.13 ± 50.62
NF-BL	60	30.36 ± 17.69	3.38 ± 1.33	86.81 ± 53.43	120.55 ± 69.38
NF-CF	60	23.78 ± 13.06	2.79 ± 1.57	63.01 ± 39.6	89.58 ± 49.84
PF-BC	80	23.09 ± 12.15	2.03 ± 1.55	59.58 ± 33.86	84.7 ± 42.88
PF-BL	90	21.17 ± 10.87	2.13 ± 1.74	55.86 ± 29.6	79.15 ± 37.73
PF-CF	70	25.81 ± 11.04	1.69 ± 1.24	76.22 ± 38.35	103.71 ± 45.68
Overall	440	25.05 ± 13.32	2.37 ± 1.54	69.16 ± 40.33	96.59 ± 50.98

The forest types included natural broad-leaved forest (NF-BL), natural coniferous forest (NF-CF), natural broadleaf-conifer mixed forest (NF-BC), planted broad-leaved forest (PF-BL), planted coniferous forest (PF-CF), and planted broadleaf-conifer mixed forest (PF-BC).

The carbon storage capacity differed significantly among the forest types (ANOVA, *P* < 0.001), and these differences were not consistent across all the carbon pools (**Figure 2**). At the total ecosystem level, natural broad-leaved forestland (NF-BL) presented the highest carbon density, which was significantly greater than that of planted broad-leaved (PF-BL) and planted broadleaf-conifer mixed (PF-BC) forests. In the tree layer, the dominance of NF-BL was notable, with its carbon density being significantly greater than that of the planted broad-leaved (PF-BL) and planted broadleaf-conifer mixed (PF-BC) forests. The pattern in the understory layer was the most distinct: NF-BL and the natural broadleaf-conifer mixed forest (NF-BC) exhibited the highest carbon densities, whereas the planted coniferous forest (PF-CF) had the lowest and formed a separate statistical group (labeled ‘d’).

Notably, the total carbon density of the planted coniferous forest (PF-CF) was significantly greater than that of the other two planted forest types. This pattern is driven primarily by its high soil organic carbon density (SOCD), which, as shown in **Figure 1**, was not significantly different from that of the natural forest types but was significantly greater than that of the planted broad-leaved forest (PF-BL, labeled ‘c’). Concurrently, the understory carbon density (UCD) of PF-CF was the lowest among all the forest types (labeled ‘d’). This distinct pattern of high soil carbon coinciding with low understory carbon suggests a unique carbon allocation strategy within PF-CF ecosystems.

### 3.2 Correlations among carbon pools and driving factors

**Figure 3:**
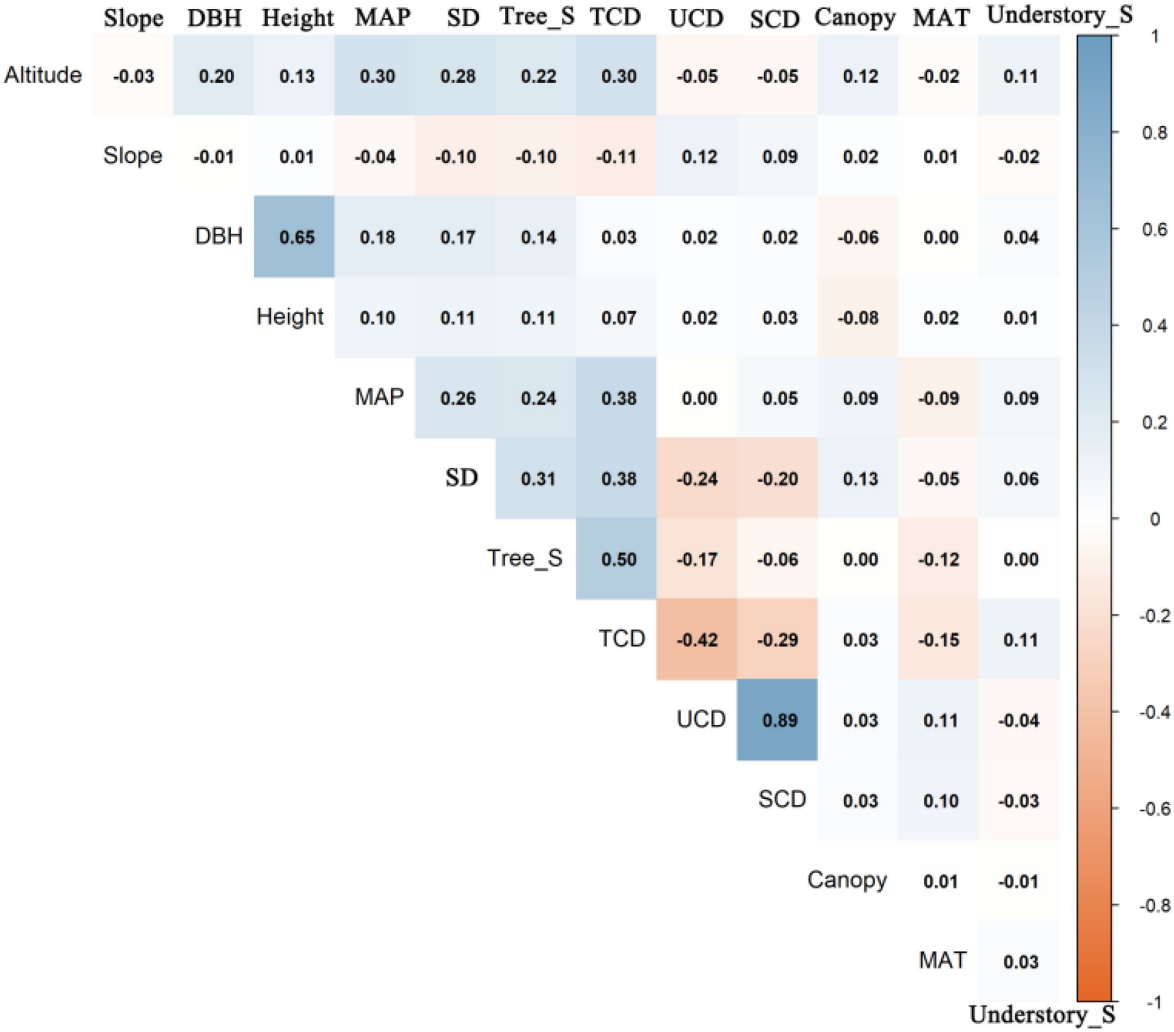
Pearson correlation matrix for carbon pools and their potential driving factors. The upper triangle displays the correlation coefficients, with the color indicating the direction and strength of the correlation (blue for positive, orange for negative). The order of the variables was determined by hierarchical clustering to visually group variables with similar correlation patterns.

Pearson’s correlation analysis revealed a trade-off in the vertical allocation of carbon, as well as a complex network of relationships dominated by stand attributes and regulated by macroenvironmental gradients (**Figure 3**). TCD was significantly negatively correlated with both the understory layer carbon density (UCD; *r* = -0.42) and the shrub layer carbon density (SCD; *r* = -0.29), confirming the strong suppressive effect of the tree canopy on understory vegetation.

Intrinsic stand attributes were the dominant factors regulating carbon allocation. Tree diversity (Tree_S_Index) was the variable most strongly and positively correlated with the TCD (*r* = 0.50), followed by stand density (Stand_Density; *r* = 0.38). However, both factors intensified the suppression of the understory, with correlation coefficients between UCD and tree diversity and stand density of -0.17 and -0.24, respectively. These findings reveal a critical management trade-off, as strategies aimed at maximizing TCD are likely to negatively impact UCD.

Topographic and climatic factors played macroregulatory roles. Both altitude (*r* = 0.30) and mean annual precipitation (MAP; *r* = 0.38) had significant positive effects on TCD. Notably, altitude was also positively correlated with stand density (*r* = 0.28) and tree diversity (*r* = 0.22), suggesting that high-altitude areas may indirectly promote TCD by supporting more mature and complex forest communities.

The analysis also revealed significant collinearity among stand structural variables, which required careful consideration for subsequent model construction. A strong positive correlation was found between the mean DBH and height (*r* = 0.65), whereas a negative correlation was observed between the DBH and Stand_Density (*r* = -0.29), reflecting the self-thinning rule. These relationships underscore the need for careful variable selection in multivariate models to avoid the confounding effects of multicollinearity.

### 3.3 Universal drivers of carbon pool variation

We used linear mixed-effects models (LMMs) to quantify the independent effects of each driving factor while controlling for differences among forest types. The fixed-effects results of the best-fit models revealed that the universal drivers of variation differed among the tree, understory, and soil carbon pools (**Table 4**).

**Table 4:** Results of the best-fit linear mixed-effects models (LMMs) showing the fixed effects of predictor variables on the carbon densities of the tree layer, understory layer, and soil organic carbon pools.

Predictor	Tree layer carbon (log)			Understory carbon (log)			Soil organic carbon (log)		
	Estimate (β)	95% CI	p value	Estimate (β)	95% CI	p value	Estimate (β)	95% CI	p value
Fixed Effects									
(Intercept)	3.12 ***	3.04–3.20	<0.001	1.13 ***	0.95–1.32	<0.001	4.08 ***	4.01–4.15	<0.001
Altitude	0.05 *	0.01–0.10	0.028	0.02	-0.01–0.05	0.273	0.04	-0.00–0.08	0.066
MAT	-0.05 *	-0.09 – -0.00	0.038	0.02	-0.01–0.05	0.195	-0.07 ***	-0.10 – -0.03	<0.001
MAP	0.06 *	0.01–0.11	0.012	0.03	-0.00–0.07	0.067	-0.02	-0.06–0.02	0.328
Stand Density	0.11 ***	0.06–0.16	<0.001	-0.06***	-0.09 – -0.02	0.001	0.03	-0.01–0.07	0.128
DBH	-0.05 *	-0.10 – -0.01	0.024	0.01	-0.02–0.04	0.636	0.05 *	0.01–0.09	0.017
Canopy	-0.03	-0.07–0.01	0.170	0.02	-0.01–0.05	0.169	0	-0.04–0.03	0.833
Tree_S	0.25 ***	0.20–0.30	<0.001	-0.01	-0.04–0.03	0.770	0.04	-0.01–0.08	0.114
Tree Layer C (scaled)				-0.22 ***	-0.30 – -0.13	<0.001	0.36 ***	0.31–0.41	<0.001
Understory C (scaled)							-0.10 ***	-0.15 – -0.06	<0.001
Random Effects									
σ²	0.20			0.10			0.15		
*τ*_00_	0.01_Type_			0.05_Type_			0.01_Type_		
*τ*_11_				0.01_Type.TCD_scaled_					
*ρ*_01_				1.00 _Type_					
N	6_Type_			6_Type_			6_Type_		
Observations	440			440			440		
Marginal R²/Conditional R²	0.361/0.383			0.351/NA			0.587/0.601		

The estimates (β) are standardized coefficients. Significance levels are denoted as follows: * *p* < 0.05, ** *p* < 0.01, and *** *p* < 0.001.

For the TCD, variation was driven primarily by stand attributes. Tree diversity (Tree_S) and stand density (Stand Density) were the strongest positive drivers (β = 0.25 and 0.11, respectively; *P* < 0.001). The altitude and MAP also exerted significant positive effects, whereas the MAT and mean DBH had significant negative effects (*P* < 0.05). With respect to the UCD, the model revealed that competitive pressure from the overstory was the primary regulatory mechanism. TCD was the sole, highly significant negative driver (β = -0.22, *P* < 0.001), and stand density also had a significant suppressive effect (β = -0.06, *P* = 0.001). With respect to the SOCD, TCD was the main positive predictor (β = 0.36, *P* < 0.001). In contrast, both UCD and MAT yielded highly significant negative effects (β = -0.10 and -0.07, respectively; *P* < 0.001).

**Figure 4:**
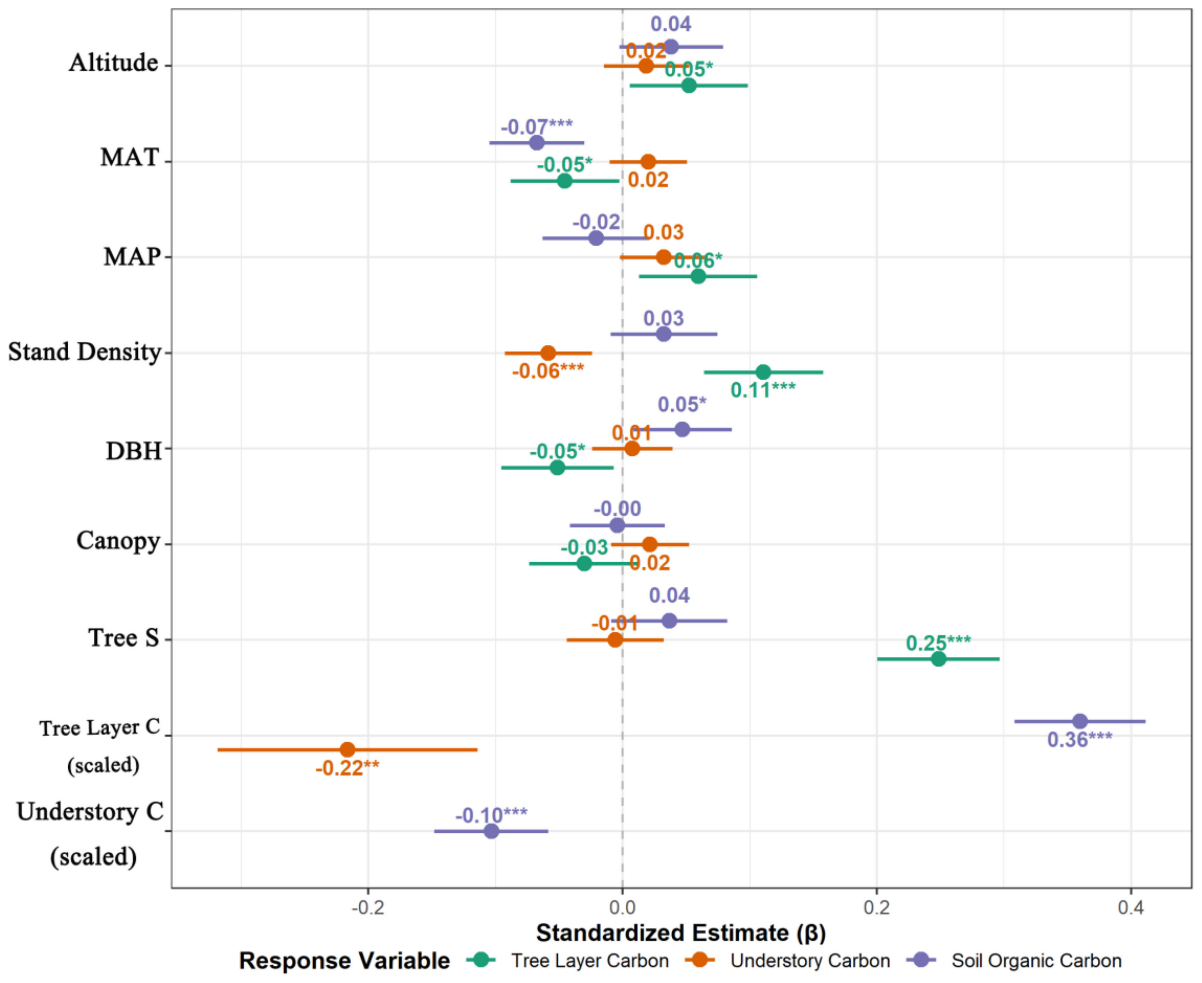
Standardized estimates (β coefficients) of the fixed effects from the best-fit linear mixed-effects models (LMMs) for the three main carbon pools. Points represent the mean estimates, and horizontal bars represent the 95% confidence intervals (CIs). Predictors with CIs that do not overlap the vertical zero line have a statistically significant effect (*p* < 0.05).

The standardized coefficients (β) and their 95% confidence intervals from the best-fit LMMs are shown in **Figure 4**, allowing for a visual comparison of the strength and direction of each driver’s effect. For tree layer carbon (green points), the effects of tree diversity and stand density were significantly positive, as their confidence intervals did not overlap the zero line. For understory carbon (orange points), the suppressive effects of Tree Layer C and stand density were significant, with their effect points located to the left of the zero line. With respect to soil carbon (purple points), Tree Layer C had the strongest positive effect, whereas Understory C had a significant negative effect, revealing the opposing effects of different aboveground strata on soil carbon accumulation. The confidence intervals for some variables, such as the canopy, stabilized the zero line, indicating that their effects were not statistically significant.

### 3.4 Context dependency of the tree–understory trade-off

We investigated the context dependency of driver effects by testing for random slope effects. The model selection results confirmed that for UCD, the model with a random slope for TCD (model UCD_4) was the best-fit model (ΔAICc = 0.00) (**Appendix Table A1**). These findings indicate that the strength of the suppressive effect of TCD on UCD, i.e., the tree–understory trade-off, differs significantly among forest types.

**Figure 5:**
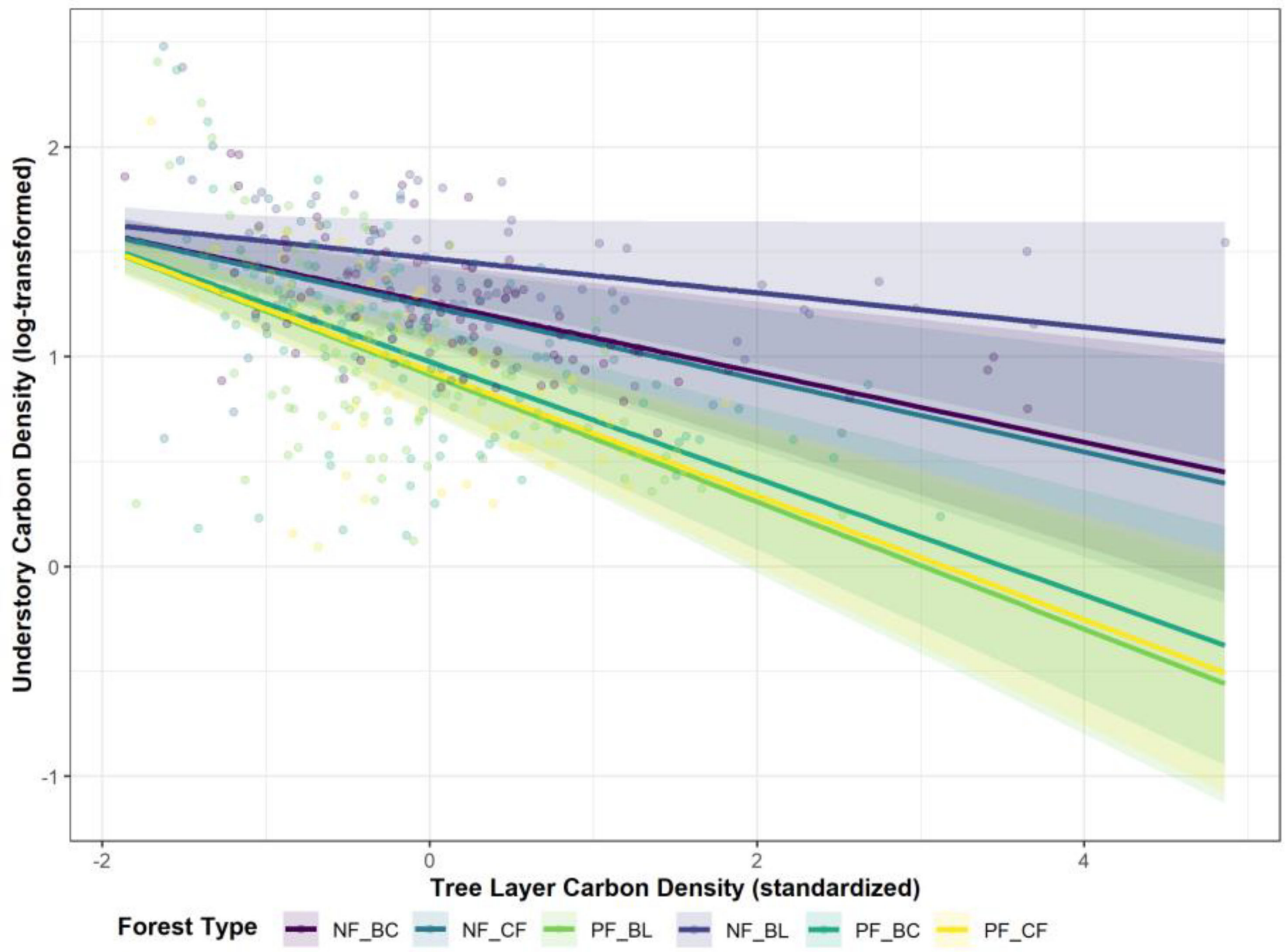
Interaction effect between tree layer carbon density (TCD) and forest type on understory carbon density (UCD). Lines represent the predicted marginal effects of TCD on the log-transformed UCD for each of the six forest types, derived from the best-fit linear mixed-effects model with a random slope for TCD (lmm_ucd_4). Shaded areas represent the 95% confidence intervals. Raw data points are plotted in the background, colored by forest type.

Visualization of this interaction reveals that the predicted regression lines for all six forest types have negative slopes, confirming that the suppression of UCD by TCD is a universal phenomenon (**Figure 5**). However, the slopes of these lines differ markedly among forest types, revealing distinct mechanisms through which natural and planted forests regulate this trade-off.

The regression lines for planted forests are generally steep, indicating strong internal trade-offs. The slopes for planted coniferous (PF-CF) and planted broad-leaved (PF-BL) forests are the steepest, implying that in these structurally simplified ecosystems, an increase in tree layer biomass leads to the most rapid decline in the understory. In contrast, the regression lines for all natural forest types are relatively gentle, with those for natural broad-leaved (NF-BL) and natural broadleaf-conifer mixed (NF-BC) forests being the shallowest. These findings demonstrate that natural forest ecosystems have a strong internal buffering capacity, allowing them to mitigate the competitive suppression from the overstory and thus weaken the tree–understory trade-off.

### 3.5 Carbon allocation pathways in natural and planted forests

**Figure 6:**
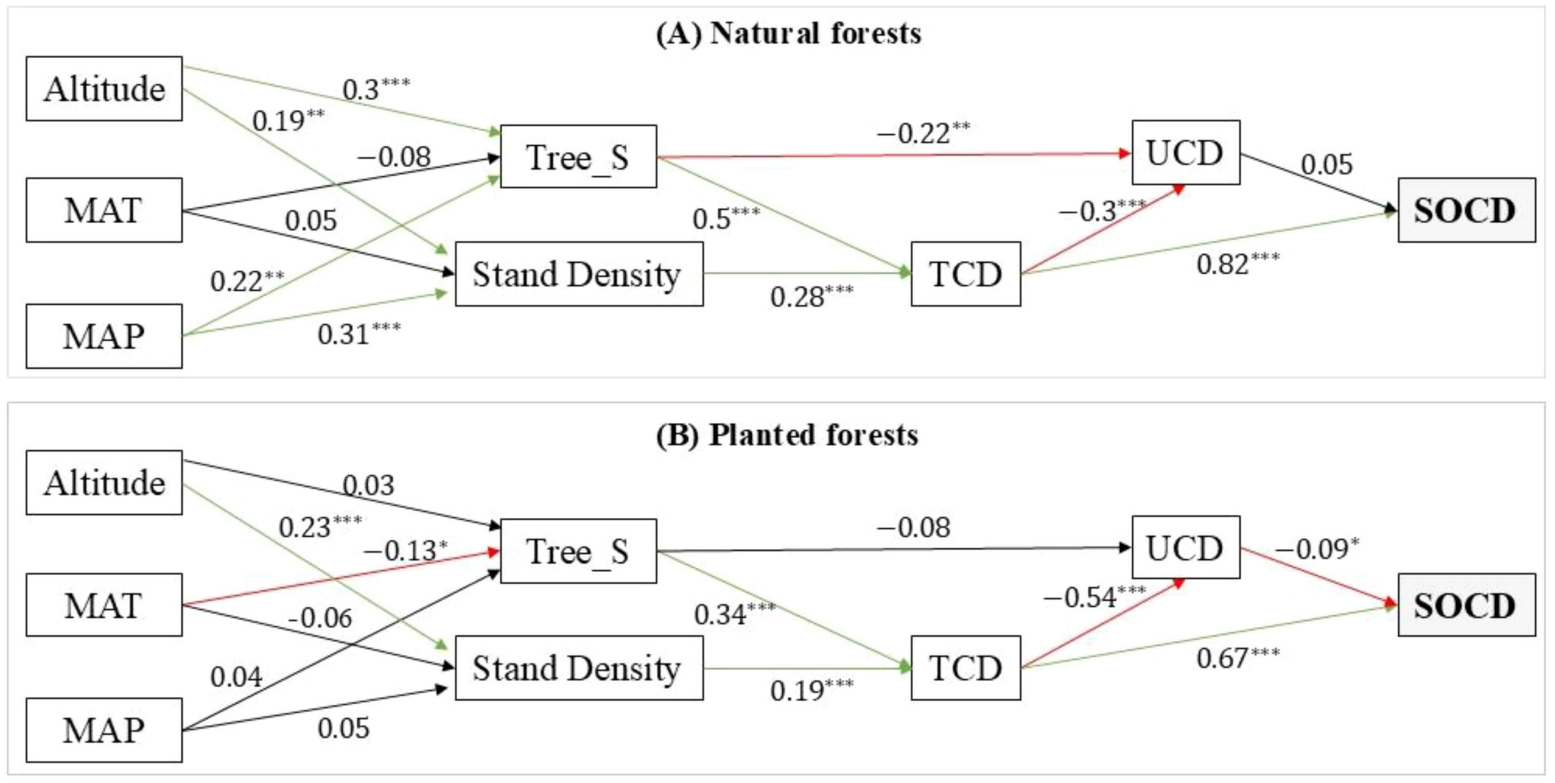
Final results of the structural equation models (SEMs) illustrating the mechanistic pathways of carbon allocation in **(A)** natural forests and **(B)** planted forests. The solid green and red arrows represent significant positive (synergistic) and negative (trade-off) paths (P < 0.05), respectively. Standardized path coefficients are shown numerically on each path.

The SEM results revealed significant differences in the carbon allocation mechanisms between natural and planted forests (**Figure 6**). Both models met the criteria for a good or acceptable fit, indicating that they effectively reproduced the relationships observed in the data (natural forests: χ² = 16.31, df = 9, *p* = 0.061, CFI = 0.986, RMSEA = 0.064; planted forests: χ² = 23.96, df = 10, *p* = 0.008, CFI = 0.960, RMSEA = 0.076).

The model for natural forests (**Figure 6A**) revealed a complex, multipathway network. Altitude, Tree_S, and Stand_Density all directly or indirectly promoted the accumulation of TCD. TCD, in turn, strongly suppressed UCD (β = -0.30). However, Tree_S played a critical buffering role: it was the strongest direct factor promoting TCD (β = 0.50) and promoting UCD through a significant direct positive pathway (β = 0.22), counteracting some of the negative impact of TCD. The increase in SOCD was driven primarily by the positive effect of TCD (β = 0.82) and was also weakly influenced by the positive effect of UCD, resulting in the formation of a synergistic pathway for carbon sequestration.

In contrast, the model for planted forests (**Figure 6B**) showed a simplified and linear system with notable trade-offs. The influence of Tree_S substantially diminished, and its direct buffering pathway to UCD was no longer significant (β = -0.08, *p* > 0.05). Consequently, the increase in the TCD driven by Stand_Density (β = 0.19) resulted in a strong suppression of UCD (β = -0.54). The system’s carbon flow was highly dependent on the single tree-to-soil pathway (TCD → SOCD, β = 0.67), lacking the multipathway regulatory mechanisms found in natural forests. Furthermore, the pathway from UCD to SOCD had a significant negative effect in planted forests (β = -0.09), in stark contrast to the positive pathway in natural forests, highlighting the simplification and potential antagonism of internal relationships.

## 4 Discussion

### 4.1 Vertical trade-offs and diversity buffering as core mechanisms underlying carbon allocation

A core finding of this study is that vertical carbon allocation in subtropical forests is dominated by asymmetric competition, but this trade-off is effectively mitigated by biodiversity. Our LMM results confirmed that an increase in tree layer carbon density had a strong and universal suppressive effect on the understory carbon pool (β = -0.22). These results are consistent with the general understanding from studies on vertical stratification in forests, which identified asymmetric competition for light as a key mechanism shaping understory community structure and productivity (DeMalach et al., 2016; Hakkenberg et al., 2020). Tall trees disproportionately acquire light energy, strongly suppressing understory vegetation through a phenomenon known as the canopy dominance effect (Schwinning and Weiner, 1998; Aschehoug et al., 2016; Rasmussen and Weiner, 2017).

However, unlike previous studies that focused primarily on describing this trade-off, our research revealed that in species-rich natural forests, tree diversity directly promotes the accumulation of understory carbon via a significant positive pathway (β = 0.22, SEM). These findings provide strong evidence that biodiversity acts as a critical buffer in these ecosystems, effectively alleviating functional trade-offs. These findings offer new empirical support for the biodiversity–ecosystem multifunctionality (BEMF) theory, which posits that high biodiversity not only enhances single functions (e.g., productivity) but also promotes synergy and reduces conflict among multiple functions (Gamfeldt et al., 2013; Mittelbach and McGill, 2019; Bai et al., 2024). Our study demonstrated that this buffering effect is a fundamental functional advantage of natural forests over planted forests.

### 4.2 Ecological explanations: niche complementarity and facilitation

Niche complementarity is a key mechanism through which diversity alleviates the tree–understory trade-off. A canopy comprising multiple tree species with different crown architectures, leaf phenologies, and physiological traits creates a heterogeneous canopy structure in both space and time (Ligot et al., 2013; Jucker et al., 2015; Martin-Blangy et al., 2022). This heterogeneity increases light penetration and the formation of sunflecks, increasing light availability in the understory and creating growth opportunities for shade-tolerant or opportunistic plants (Loreau and Hector, 2001). Our SEM analysis confirmed that this diversity-mediated buffering pathway is absent in even-aged, single-species planted forests, which aligns with the view of plantations as simplified ecosystems with linear and vulnerable ecological processes (Carnus et al., 2006; Brockerhoff et al., 2008; Pawson et al., 2013).

Facilitation is another key mechanism. In addition to light availability, nutrient and water availability are important. Some tree species can increase soil nutrient availability through nitrogen fixation or specific litter decomposition processes, which can positively affect the growth of other trees and understory plants (Aleixo et al., 2020; Didur and Shevchuk, 2020). Similarly, diverse root systems can effectively acquire water from different soil layers and may indirectly benefit understory plants through hydraulic lift (Yi et al., 2021). Although our study did not directly measure these processes, our path analysis results indicate that protecting and restoring tree layer biodiversity is a core pathway for maximizing the carbon sink function of an ecosystem by enhancing niche complementarity and facilitating a reduction in internal competition.

### 4.3 Divergent carbon allocation mechanisms and implications for sustainable forest management

Our SEM analysis revealed that forest origin (natural vs. planted) fundamentally reshaped the carbon allocation mechanisms in the various ecosystems. Natural forests exhibited a complex network with multiple pathways and feedback regulations, whereas planted forests displayed a linear, simplified system with pronounced trade-offs. As our models showed, the diversity-mediated buffering pathway in natural forests significantly weakened the suppression of the understory by the tree layer. In contrast, the absence of this buffering pathway in planted forests suggests that the increased tree layer growth driven by density was almost entirely converted into strong suppression of the understory, leading to a sharp internal trade-off.

These findings have clear implications for forest management strategies aimed at achieving carbon neutrality. First, the carbon sink potential of all the forest types is not equal. Our data indicate that conserving and restoring natural forests is the most robust strategy for maximizing regional carbon storage. Second, for vast areas of planted forests, management should shift from solely maximizing timber yield to enhancing ecosystem multifunctionality. Our research shows that adopting close-to-nature silviculture practices, such as establishing mixed-species stands and optimizing stand structure, can rebuild the complexity and functional redundancy of natural forests, which is key to enhancing multiple ecosystem services, including carbon sequestration (Weetman, 1987; Bauhus et al., 2009).

Furthermore, a striking finding from our SEM analysis is that the effect of the understory carbon pool on soil carbon is directionally opposite in the two forest types (positive in natural forests, and negative in planted forests). While our study cannot elucidate the underlying mechanisms, these results point to fundamental differences in belowground processes. This difference may be linked to several factors, including differences in root traits, litter quality, and their subsequent effects on soil microbial communities (Kuzyakov, 2010). In natural forests, the positive pathway suggests that diverse, high-quality inputs from the understory promote the formation of stable soil organic matter. In contrast, the negative pathway in planted forests could be attributed to mechanisms such as a microbial “priming effect” where the input of low-quality (e.g., high C:N ratio) litter stimulates microbes to “mine” for nutrients by decomposing pre-existing, stable soil organic carbon. These findings serve as a critical reminder that when assessing the carbon sink function of plantations, the complex and sometimes counterintuitive interactions within the belowground system must be considered to avoid misjudgments of the ecosystem’s carbon balance.

Beyond these general principles, a particularly noteworthy result is that the SOCD in planted coniferous forests (PF-CF) was significantly greater than that in other planted forests and was statistically comparable to that in natural forests. Our analysis revealed that this pattern coincides with the extremely low UCD in PF-CF, which strongly suggests that the functional traits of the dominant tree species may, under certain conditions, be key factors regulating soil carbon accumulation. Two complementary mechanisms could underlie this phenomenon. First, compared with broadleaf litter, coniferous litter generally decomposes more slowly, which may facilitate the accumulation of organic matter in the soil (Vesterdal et al., 2013). Second, the lack of understory vegetation in PF-CF might reduce the priming effect that could otherwise be induced by understory root exudates, thereby decreasing the decomposition of pre-existing, stable soil organic carbon (Kuzyakov, 2010). These findings caution that when assessing the carbon sink function of plantations, the functional traits of the dominant tree species and their unique effects on belowground processes must be considered to avoid misjudgments of the ecosystem’s overall carbon balance.

### 4.4 Limitations and future directions

Although this study provides new insights into the mechanisms of carbon allocation in subtropical forests, we acknowledge several limitations. First, our study is a large-scale investigation based on space-for-time substitution. While it reveals clear patterns and relationships, it cannot directly capture the dynamic evolution of these relationships over time. Consistent with Lindenmayer et al. (2013), we believe that establishing long-term forest monitoring transects with different management regimes will be crucial for validating and deepening the mechanisms discovered in this study.

Second, our structural equation models, while revealing clear causal pathways, still contain black boxes. For example, we inferred that tree diversity mitigates the tree–understory trade-off by improving the microenvironment, but we did not directly measure these process variables. Future research should integrate macroscopic ecosystem patterns with microscopic process measurements. For instance, hemispherical photography can be used to quantify the heterogeneity of understory light (Konôpka et al., 2016), and litter decomposition experiments can be used to assess the effects of different tree species on nutrient cycling (Gessner et al., 2010).

Third, our carbon accounting framework has a defined boundary that warrants discussion. This study focused on the carbon stocks in aboveground living vegetation and mineral soil, and therefore did not explicitly quantify the carbon stored in coarse roots (> 2 mm) of the tree layer. While fine root carbon is implicitly included in the SOCD measurements, the exclusion of coarse root biomass means that our estimates of the total ecosystem carbon density (TECD) are inherently conservative. Given that the root-to-shoot ratio can vary significantly among species and environments, future research integrating robust root biomass models or nondestructive techniques (e.g., ground-penetrating radar) would provide a more holistic understanding of the total ecosystem carbon budget and its allocation between above- and belowground components.

Finally, our inferences about the priming effect and multicollinearity handling also point to future research directions. The potential for a negative priming effect from the understory in planted forests requires more direct validation using advanced isotope labeling techniques (e.g., ¹³C or ¹^4^C pulse labeling) to trace carbon flows. Furthermore, while our variable selection approach was chosen to preserve the interpretability of individual predictors, future studies focused on predictive performance could explore dimensionality reduction techniques such as principal component analysis (PCA) or machine-learning algorithms to address multicollinearity in a different manner.

## 5 Conclusions

This study revealed the allocation patterns and driving mechanisms of multilevel carbon pools in subtropical forests. We found that the forest ecosystem carbon pool is an integrated system governed by both synergies and trade-offs. The growth of the tree layer has a strong positive synergistic effect on the soil carbon pool, but it also significantly suppresses understory vegetation through asymmetric competition, creating a core vertical trade-off. These interactions strongly depend on the forest management model, with biodiversity playing a critical buffering role. In structurally complex natural forests, tree diversity effectively mitigates the suppression of the understory through niche complementarity and facilitation. In contrast, the absence of this buffering pathway in structurally simple planted forests leads to pronounced internal trade-offs.

Therefore, we propose that a forest’s carbon sink function is determined not only by its total biomass but also by the carbon allocation mechanisms dictated by its structural complexity and functional diversity. Maximizing the carbon sequestration potential of forests requires optimizing the structure and function of the entire ecosystem rather than simply pursuing the growth of a single pool. Our findings contribute to the growing consensus that ecosystem functions constitute emergent properties of the complex interactions among their components and that biodiversity is key to maintaining the stability and synergy of these functions.

## Data Availability

The raw data supporting the conclusions of this article will be made available by the authors, without undue reservation.
