# Supplementary material for: Synergies and trade-offs in forest carbon pools: separating universal drivers from forest type-specific controls
Source: Front Plant Sci. 2026 Jan 6;16:1728262. doi: 10.3389/fpls.2025.1728262 (PMC12815779; doi:10.3389/fpls.2025.1728262)
Supplement: Supplementary file 1 [file Table1.docx]

# **Appendix A**

Figure A1: Location of the study area and distribution of the 440 sampling plots. The main panel shows the spatial distribution of the six forest types overlaid on an altitude map of the south-central region of Hunan Province, China. The top-left inset indicates the location of the study region within Hunan Province. The bottom-left inset provides the legend for the forest types and the altitude gradient. Forest type abbreviations are as follows: NF-BC (Natural Broadleaf-Conifer Mixed Forest), NF-BL (Natural Broad-leaved Forest), NF-CF (Natural Coniferous Forest), PF-BC (Planted Broadleaf-Conifer Mixed Forest), PF-BL (Planted Broad-leaved Forest), and PF-CF (Planted Coniferous Forest).


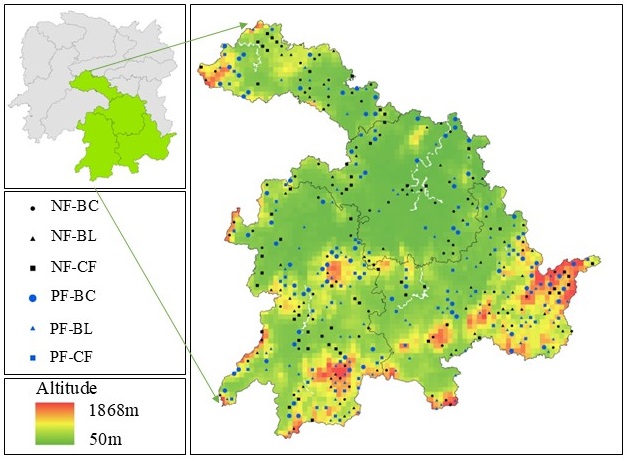


Table A1: Model selection results of the linear mixed-effects models (LMMs) for predicting the carbon densities of different ecosystem pools. Models were ranked based on the Akaike information criterion corrected for small sample sizes (AICc). The best-fit model for each response variable is indicated by the lowest AICc value (ΔAICc = 0.00).

| *Response.Variable* | *Model.ID* | *Model.Structure* | *k* | *AICc* | *ΔAICc* | *Rank* |
| --- | --- | --- | --- | --- | --- | --- |
| SOCD | 1 | Fixed Effects + (1 \| Type) | 12 | 499.30 | 0.00 | 1 |
| SOCD | 2 | Fixed Effects + (1 + Stand Density \| Type) | 14 | 502.71 | 3.41 | 3 |
| SOCD | 3 | Fixed Effects + (1 + Tree_S \| Type) | 14 | 503.44 | 4.14 | 4 |
| SOCD | 4 | Fixed Effects + (1 + TCD_scaled \| Type) | 14 | 499.55 | 0.25 | 2 |
| TCD | 1 | Fixed Effects + (1 \| Type) | 10 | 611.32 | 0.00 | 1 |
| TCD | 2 | Fixed Effects + (1 + Stand Density \| Type) | 12 | 615.43 | 4.10 | 4 |
| TCD | 3 | Fixed Effects + (1 + DBH \| Type) | 12 | 613.44 | 2.11 | 2 |
| TCD | 4 | Fixed Effects + (1 + Tree_S \| Type) | 12 | 614.63 | 3.31 | 3 |
| UCD | 1 | Fixed Effects + (1 \| Type) | 11 | 364.18 | 23.97 | 3 |
| UCD | 2 | Fixed Effects + (1 + Stand Density \| Type) | 13 | 360.46 | 20.25 | 2 |
| UCD | 3 | Fixed Effects + (1 + Tree_S \| Type) | 13 | 366.73 | 26.52 | 4 |
| UCD | 4 | Fixed Effects + (1 + TCD_scaled \| Type) | 13 | 340.21 | 0.00 | 1 |
